# Association between cytokine gene polymorphisms and tuberculosis in a Chinese population in Shanghai: a case–control study

**DOI:** 10.1186/s12865-015-0071-6

**Published:** 2015-02-22

**Authors:** Yi Hu, Linlin Wu, Dange Li, Qi Zhao, Weili Jiang, Biao Xu

**Affiliations:** Department of Epidemiology, School of Public Health, Fudan University, 138 Yi Xue Yuan Road, Shanghai, 200032 China; Key Laboratory of Public Health Safty (Fudan University), Ministry of Education, Shanghai, China

**Keywords:** Tuberculosis, Latent tuberculosis infection, Cytokine, Polymorphism, Association

## Abstract

**Background:**

Polymorphisms in cytokine genes are known to influence cytokine levels, which may influence susceptibility to tuberculosis (TB) infection and disease. Differences in cytokine expression probably determine whether TB progresses, resolves, or becomes latent. In particular, the balance between the Th1 and Th2 cytokine responses influences the expression of disease in individuals with pulmonary TB (PTB). We performed a case–control study of 120 patients diagnosed with PTB, 240 with latent TB infection (LTBI), and 480 healthy controls (HC), to explore the association between polymorphisms in cytokine genes and a predisposition to *Mycobacterium tuberculosis* infection and TB disease.

**Results:**

A single-gene analysis showed a dominant association between the AA genotype or A allele at nucleotide −874 of the interferon γ (IFN-γ) gene and LTBI. The A allele at nucleotide −1082 of the interleukin 10 (IL-10) gene was significantly more common in PTB patients than in LTBI subjects. Moreover, the polymorphisms at *IFN-γ* −874 and *IL10* − 1082 were associated with protein levels of IFN-γ and IL-10, respectively, in the PTB group. The genotype frequencies of other polymorphisms did not differ between the PTB patients, LTBI and HC subjects. Furthermore, combinations of polymorphisms with *IFN-γ* −874 were associated with LTBI, whereas combinations with *IL10* − 1082 were more likely associated with PTB.

**Conclusions:**

There are positive associations between the *IFN-γ* −874 polymorphism and TB and between the *IL10* − 1082 polymorphism and LTBI. Our data provide genetic evidence of the multiple disease hypothesis that many cytokine genes are involved in TB susceptibility.

## Background

Tuberculosis (TB) remains to be a leading cause of morbidity and mortality in developing countries, and the incidence of the disease is increasing in developed countries [1]. One third of the world’s population is infected by *Mycobacterium tuberculosis* (Mtb), but only about 5% of infected individuals develop the disease within the first year of infection and another 5% develop the disease later in life. Candidate gene and association studies have identified various host genetic factors that play significant roles in susceptibility to TB [2,3]. Identifying the host genes responsible for susceptibility and resistance to TB may lead towards better understanding of the pathogenesis of TB and development of prophylactic or treatment strategies.

The cytokines produced at the site of disease after the interaction between T lymphocytes and infected macrophages are essential for the pathogenesis of TB [4]. The course of Mtb infection is regulated by two distinct T-cell cytokine patterns. T-helper 1 (Th1) cytokines, interleukin(*IL*)-2 and interferon γ (IFN-γ), are associated with resistance to infection, whereas T-helper 2 (Th2) cytokines, *IL-4* and *IL-13*, are associated with progressive disease [5]. Polymorphisms in several cytokine genes have been described and shown to influence gene transcription, leading to inter individual variations in cytokine production [6]. Cytokine gene polymorphisms have been shown to be involved in the susceptibility, the severity and clinical outcomes of several diseases, including infectious diseases [7]. A single nucleotide polymorphism (SNP) located in the first intron of human the *IFN-γ* gene (+874 T⁄ A) putatively influences the secretion of the cytokine, with an impact on the outcomes of infections. This polymorphism displays variable associations with TB disease susceptibility and severity [8] in some ethnic populations but not in others [9]. A specific nucleotide polymorphism within the promoter region of *IL-10* (1082A⁄G) is also highly variable. This polymorphism alters the level of this cytokine and possibly the Th1/Th2 balance, with major implications for TB infection [6]. Data on the association between the 1082G/A SNP of *IL-10* and TB are also highly variable. Among the proinflammatory cytokines, tumor necrosis factor α (*TNF-α*) SNP −308 is associated with either TB susceptibility or severity [10]. Furthermore, because the immune interactions that determine TB disease susceptibility and severity are complex, multigene combinations may give more meaningful information than single SNPs in determining disease outcomes [11].

Most studies have not differentiated TB disease according to its natural history. Therefore, it is difficult to evaluate the differences in their results. Our study groups were stratified into latent TB infection (LTBI) and active TB disease. We investigated the influence of *IFN-γ*, *TNF-α*, and interleukin cytokine gene polymorphisms on the susceptibility of individuals to LTBI and TB disease in terms of the cytokine levels expressed and their associations with *ex vivo* stimulate cytokine and pulmonary TB in a population from Shanghai. We also analyzed combinations of cytokine gene polymorphisms.

## Results

### Demographic characteristics of the study groups

The study subject groups consisted of 120 newly diagnosed PTB patients, 240 subjects with LTBI, and 480 healthy control (HC) subjects. The mean ages and sex distributions of the patients and controls did not differ. The Bacille Calmette– Guérin (BCG) scar was observed 87.5% of PTB patients, 93.8% of the LTBI group, and 94.0% of HC subjects (Table 1).

**Table 1:** **Baseline information on the study groups**

	**HC (n = 480)**	**LTBI (n = 240)**	**PTB (n = 120)**	**χ** ^**2**^ **/F**	**p value**
Age (mean ± SD)	41.0 ± 19.9	43.0 ± 18.1	46.0 ± 12.2	0.141	0.234
Sex (male) N(%)	294(61.2)	147(61.2)	74(61.7)	0.008	0.996
BCG scar N(%)	451(94.0)	225(93.8)	105(87.5)	6.440	0.040*

Notes: SD, standard deviation; HC, healthy controls; LTBI, subjects with latent tuberculosis infection; PTB, patients with pulmonary tuberculosis; BCG, Bacillus Calmette–Guérin vaccine.

*p<0.05.

### Associations between Th1 cytokine polymorphisms and TB susceptibility

**Figure 1:**
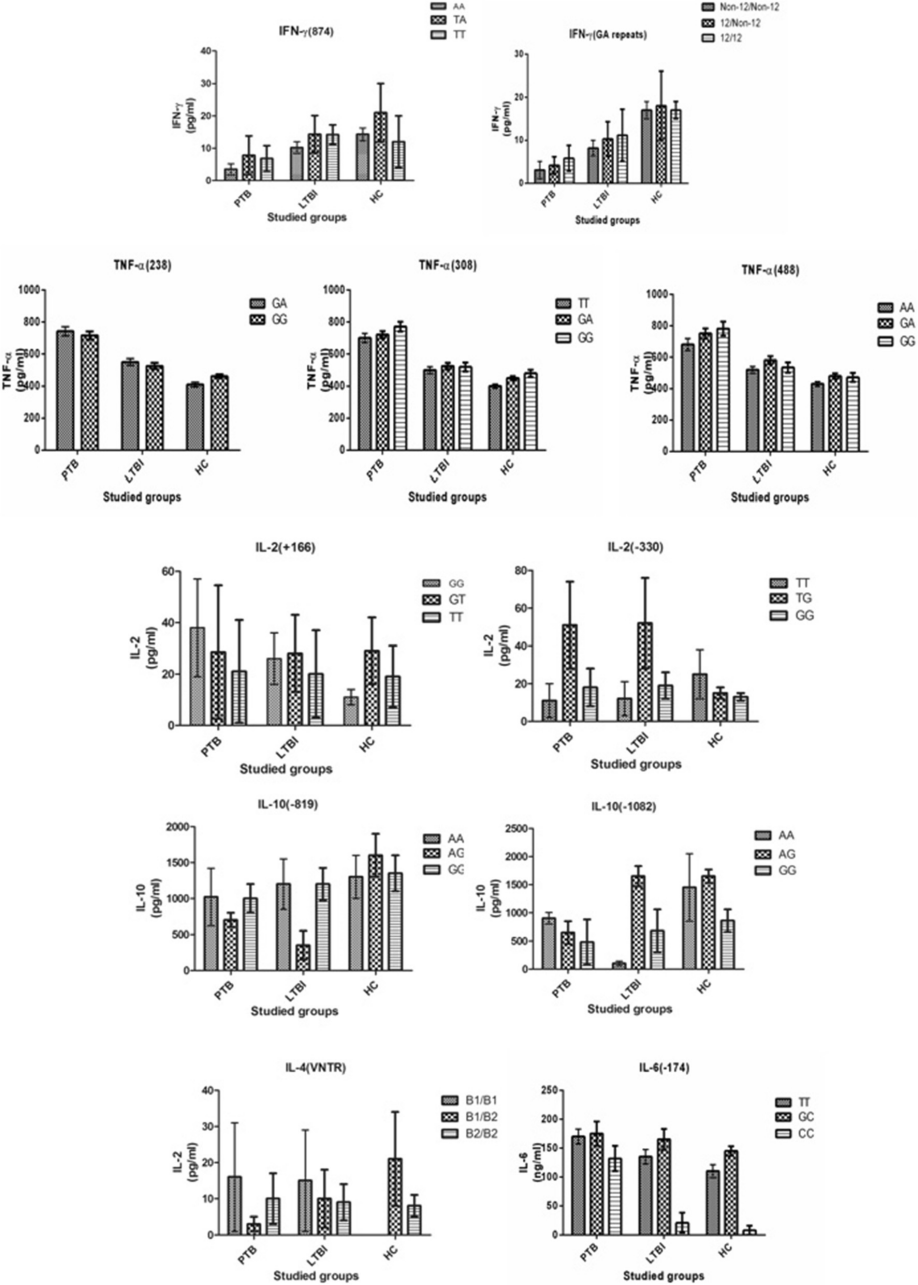
**Influence of cytokine polymorphism on culture filtrate (CFA) of M.tuberculosis-induced cytokine production in healthy controls, LTBI and PTB patients.**

The genotype distributions of the *IFN-γ* −874A/T SNP and CA repeat did not deviate from Hardy–Weinberg equilibrium in any subjects. At the *IFN-γ* T874A polymorphic site, the frequency of the A allele was 1.30-fold higher in the LTBI than in the HC subjects (OR = 1.30; 95% CI: 1.020–1.664). The LTBI group had a significantly higher proportion of the AA genotype than the healthy group (OR = 1.39; 95% CI: 1.011–1.930). However, the PTB and LTBI groups had very similar frequency distributions for the other −874A/T genotypes and alleles. We identified 10 alleles for the CA repeat polymorphism (ranging from 10 to 19 CA repeats). The 12, 13, and 15 CA repeats were found to be the most common alleles in the study groups. The three groups displayed similar distributions of the CA repeats, although the TB group contained a significantly higher proportion of the 12-repeat allele in the LTBI group (OR = 1.71; 95% CI: 1.074–2.728). A haplotype analysis revealed no significant differences in the *IFN-γ* A-12 haplotype frequency between the three groups. The *in vitro* production of IFN-γ correlated with the *IFN-γ* −874 polymorphism. IFN-γ levels were significantly lower in the LTBI and PTB groups than in the HC group (*p* = 0.001; Table 2, Figure 1).

**Table 2:** **Allele, genotype, and haplotype of Th1, Th2, and Th17 cytokine polymorphisms signficantly associated with LTBI and TB**

		**HC**	**LTBI**	**PTB**	**LTBI.VS.HCS**		**PTB.vs.LTBI**	
		**N(%)**	**N(%)**	**N(%)**	**OR(95%CI)**	**p value**	**OR(95%CI)**	**p value**
IFN-r	Genotypes							
+874(A/T)	AA	212(44.2)	126(52.5)	74(61.7)	1.39(1.011–1.930)	0.035*	1.46(0.910–2.337)	0.099
	AT	201(41.9)	88(36.7)	36(30.0)	0.80(0.576–1.119)	0.131	0.74(0.448–1.213)	0.210
	TT	67(14.0)	26(10.8)	10(8.3)	0.75(0.443–1.235)	0.171	0.75(0.311–1.676)	0.456
	Alleles							
	A	625(65.1)	340(70.8)	184(76.7)	1.30(1.020–1.664)	0.029*	1.35(0.934–1.974)	0.097
	T	335(34.9)	140(29.2)	56(23.3)	0.77(0.601–0.980)	0.029*	0.74(0.507–1.071)	0.097
CA repeats	Alleles							
	12	641(66.8)	340(70.8)	186(77.5)	1.21(0.946–1.547)	0.119	1.71(1.074–2.728)	0.017*
	Non-12	319(33.2)	140(29.2)	54(22.5)	0.83(0.647–1.057)	0.119	0.58(0.367–0.931)	0.017*
Haplotypes								
(+874 and CA repeats)	A-12	167(17.4)	103(21.5)	60(25.0)	1.30(0.975–1.721)	0.063	1.22(0.847–1.757)	0.284
	T-12	174(18.1)	75(15.6)	30(12.5)	0.84(0.613–1.134)	0.237	0.77(0.472–1.238)	0.263
	A-non12	324(33.8)	175(36.5)	98(40.8)	1.13(0.889–1.425)	0.309	1.20(0.864–1.672)	0.254
	T-non12	295(30.7)	127(26.5)	52(21.7)	0.81(0.629–1.043)	0.093	0.77(0.521–1.126)	0.161
TNF-α	Haplotypes							
(238,308,488)	A-A-A	1(0.2)	1(0.4)	0(0)	1.996(0.124–32.047)	1.000	–	-
	A-A-G	205(42.7)	102(42.5)	0(0)	0.984(0.720–1.346)	0.922	0.54(0.477–0.599)	0.000^*^
	A-G-A	0(0)	0(0)	43(35.8)	–		4.12(3.390–5.000)	0.000^*^
	A-G-G	20(4.2)	10(4.2)	12(10.0)	0.996(0.459–2.162)	0.880	2.56(1.071–6.099)	0.029^*^
	G-A-A	1(0.2)	1(0.4)	0(0)	1.996(0.124–32.047)	1.000	–	
	G-A-G	13(2.7)	7(2.9)	53(44.2)	1.075(0.423–2.730)	0.880	26.33(11.439–60.61)	0.000*
	G-G-A	210(43.8)	105(43.8)	5(4.2)	0.993(0.727–1.356)	0.963	0.06(0.022–0.142)	0.000*
	G-G-G	30(6.3)	15(6.3)	7(5.8)	0.996(0.525–1.888)	0.989	0.93(0.368–2.344)	0.876
IL2	Genotype							
−330(T/G)	GG	44(9.2)	29(12.1)	30(25.0)	1.210(0,742–1.969)	0.445	2.43(1.376–4.275)	0.002*
	GT	256(53.3)	104(43.3)	56(46.7)	0.669(0.489–0.914)	0.011^*^	1.14(0.737–1.777)	0.549
	TT	180(37.5)	107(44.6)	34(28.3)	1.34(0.966–1.858)	0.067	0.49(0.297–0.806)*	0.003*
	Alleles							
	G	344(35.8)	162(33.8)	116(48.3)	0.91(0.724–1.149)	0.435	1.84(1.339–2.519)	0.000*
	T	616(64.2)	318(66.2)	124(51.7)	1.10(0.865–1.391)	0.435	0.545(0.392–0.757)*	0.000*
−330 and −160	Haplotypes							
	G-G	205(21.4)	97(20.3)	48(20.1)	0.93(0.703–1.232)	0.615	0.99(0.655–1.475)	0.948
	G-T	99(10.3)	68(14.1)	32(13.5)	1.44(1.015–2.021)	0.031*	0.93(0.573–1.493)	0.761
	T-G	339(41.6)	95(39.5)	95(39.4)	0.45(0.344–0.590)	0.000*	2.66(1.856–3.793)	0.000*
	T-T	317(26.7)	220(26.2)	65(27)	1.72(1.362–2.161)	0.000*	0.44(0.308–0.622)	0.000*
IL-12	genotypes							
−1188(A/C)	AA	270(56.2)	162(67.5)	80(66.7)	1.62(1.154–2.269)	0.004*	0.96(0.590–1.582)	0.874
	AC	177(36.9)	65(27.1)	33(27.5)	0.66(0.460–0.933)	0.009*	1.02(0.603–1.712)	0.933
	CC	33(6.9)	13(5.4)	7(5.8)	0.78(0.367–1.55)	0.451	1.08(0.355–3.011)	0.871
	Alleles							
	A	717(74.7)	389(81.0)	193(80.4)	1.49(1.097–1.922)	0.007*	0.96(0.639–1.456)	0.841
	C	243(25.3)	91(19.0)	47(19.6)	0.69(0.520–0.911)	0.007*	1.04(0.687–1.565)	0.841
IL-10	Genotypes							
−1082(A/G)	AA	262(54.6)	136(56.7)	82(68.3)	1.09(0.787–1.506)	0.596	1.65(1.016–2.699)	0.033*
	AG	196(40.8)	86(35.8)	34(28.3)	0.81(0.579–1.128)	0.195	0.71(0.425–1.168)	0.155
	GG	22(4.6)	18(7.5)	4(3.4)	1.69(0.834–3.368)	0.107	0.43(0.103–1.335)	0.120
−1082 and −819	Haplotypes							
	A-C	205(21.4)	116(24.2)	58(24.2)	1.17(0.896–1.533)	0.235	0.24(0.683–1.456)	1.000
	A-T	100(10.4)	174(36.2)	95(39.6)	4.95(3.707–6.608)	0.000^*^	1.15(0.826–1.604)	0.383
	G-C	400(41.7)	56(11.7)	26(10.8)	0.19(0.134–0.254)	0.000^*^	0.91(0.533–1.525)	0.740
	G-T	255(26.6)	134(27.9)	61(25.4)	1.07(0.830–1.378)	0.601	0.88(0.607–1.268)	0.477
IL-6	Alleles							
−174(G/C)	G	674(70.2)	363(75.6)	182(75.8)	1.32(1.017–1.707)	0.031^*^	1.01(0.695–1.481)	0.951
	C	286(29.8)	117(24.4)	58(24.2)	0.76(0.586–0.982)	0.031^*^	0.99(0.675–1.439)	0.951
	genotypes							
	GG	247(51.5)	131(54.6)	66(55.0)	1.03(0.749–1.429)	0.429	1.02(0.640–1.620)	0.940
	GC	180(37.5)	101(42.1)	50(41.7)	1.21(0.871–1.681)	0.235	0.98(0.614–1.569)	0.940
	CC	53(11.0)	8(3.3)	4(3.3)	0.36(0.144–0.793)	0.000^*^	1.00(0.216–3.826)	1.000

Notes: HC, healthy controls; LTBI, subjects with latent tuberculosis infection; PTB, patients with pulmonary tuberculosis; INF-γ, interferon γ; TNF-α, tumor necrosis factor α; IL-2, interleukin 1; IL-12, interleukin 12; IL-10, interleukin 10; IL-4, interleukin 4; IL-6, interleukin 6.

*p < 0.05.

In *TNF-α*, the frequencies of the *TNF-α* +488A, −238A, and −308A alleles were 6.5%, 3.3%, and 4.7% in the HC group, 7.1%, 3.3%, and 5.2% in the LTBI group, respectively, and 10.0%, 4.6%, and 10.8% in the PTB group, respectively. The number of individuals homozygous for +488A was low and there were no homozygotes for −238A or −308A in the PTB group. No significant differences in the frequencies of the three polymorphic variants of *TNF-α* were observed between the healthy control and LTBI groups or between the LTBI and PTB groups. The haplotype frequencies were similar between the three groups. The levels of TNF-α were not related to the *TNF-α* polymorphisms and were similar between the three groups (Table 2, Figure 1).

At the −330 SNP of *IL-2*, a significant negative association was evident between the TT genotype and the PTB group compared with the LTBI group (28.3% vs 44.6%, OR = 0.49, 95% CI: 0.297–0.806), whereas the G allele was significantly overrepresented in the PTB group. A trend towards a reduced IL-2 response was observed with the −330 GG genotype compared with the TG and TT genotypes in culture filtrate (CFA)-stimulated cultures of peripheral blood mononuclear cells (PBMCs) from HC subjects (*p* = 0.35). No significant differences in *IL-2* levels were observed between the variant genotypes of the *IL2* polymorphism in the HC or the LTBI groups (Figure 1).

### Associations between Th2 cytokine polymorphisms and TB susceptibility

Homozygosity for the *IL-10* − 1082A allele occurred at a higher frequency in the PTB group than in the LTBI group (OR = 1.61, 95% CI: 1.071–2.438), indicating a significant association between TB and the *IL-10* − 1082 AA genotype (OR = 1.65, 95% CI: 1.061–2.699). No statistically significant difference was observed in the distributions of the *IL-10* − 1082 genotypes in the LTBI and HC groups (*p* > 0.05). The distributions of the *IL-10* − 819 genotypes were similar in the PTB patients, LTBI and HC subjects (*p* > 0.05). There was no linkage between the −1082A/G and −819C/T *IL10* polymorphisms. The frequency of the *IL-10* − 1082G/−819C haplotype was lower in the LTBI subjects than in the HC (11.6% vs 41.6%, respectively; OR = 0.19, 95% CI: 0.134–0.254), whereas the frequency of the *IL-10* − 1082 A/−819 T haplotype was higher (36.2% vs 10.3%, respectively; OR = 4.95, 95% CI: 3.707–6.608). However, the haplotype distributions did not differ significantly between the LTBI and PTB subjects and the *IL-10* levels did not differ among the variant genotypes of the *IL-10* gene polymorphisms in the two groups (Table 3).

**Table 3:** **Association between combinations of cytokine gene polymorphisms and susceptibility to LTBI and PTB**

	**INF-γ**	**TNF-a**	**IL-10**	**IL-2**	**HC**	**LTBI**	**PTB**	**LTBI.vs.Health**	**PTB.vs.LTBI**
	**874**	**−308**	**−1082**	**−330**				**OR(95%CI)**	**OR(95%CI)**
1	A	A	A	G	56	30	12	1.07(0.657-1.734)	0.79(0361–1.624)
2	A	A	A	T	113	47	43	0.81(0.555-1.178)	2.01(1.252-3.219)*
3	A	A	G	T	50	41	5	1.70(1.079-2.665)*	0.23(0.069-0.588)
4	A	G	A	G	70	32	22	9.25(5.300-15.932)*	1.41(1.062-2.575)*
5	A	G	A	T	105	67	46	1.32(0.936-1.855)	1.46(0.994-2.248)
6	A	G	G	G	29	28	7	1.98(1.125-3.505)*	0.48(0.176-1.159)
7	A	G	G	T	44	26	11	1.19(0.695-2.009)	0.84(0.367-1.795)
8	T	A	A	G	29	20	9	2.47(1.300-4.599)*	0.89(0.353-2.101)
9	T	A	A	T	64	32	11	1.00(0.623-1.578)	0.67(0.300-1.400)
10	T	A	G	G	35	11	7	0.62(0.281-1.263)	0.33(0.107-1.945)
11	T	A	G	T	71	30	8	0.83(0.518-1.319)	0.52(0.202-1.179)
12	T	G	A	G	24	19	9	1.61(0.823-3.093)	0.95(0.371-2.236)
13	T	G	A	T	85	43	17	1.01(0.673-1.508)	0.77(0.405-1.425)
14	T	G	G	G	38	20	6	1.05(0.574-1.884)	0.59(0.191-1.550)
15	T	G	G	T	124	28	16	0.42(0.263-0.645)	1.15(0.570-2.259)

Notes: HC, healthy controls; LTBI, subjects with latent tuberculosis infection; PTB, patients with pulmonary tuberculosis; INF-γ, interferon γ; TNF-α, tumor necrosis factor α; IL-10, interleukin 10; IL-2, interleukin 2.

*p < 0.05.

The allele and genotype frequencies of the *IL-4* variable number tandem repeat (VNTR) polymorphism did not differ between the HC subjects and the PTB patients. The *IL-4* response was only detectable in a few subjects. In the HC subjects, the *IL-4* levels did not differ among the variant genotypes of the *IL-4* VNTR polymorphism. However, among the PTB patients, there was a trend towards an increased *IL-4* response in patients with the B2/B1 genotype compared with the B2/B2 genotype under stimulated and unstimulated conditions (CFA, *p* = 0.066; Table 2, Figure 1).

### Associations between the Th17 cytokine polymorphism and TB susceptibility

For the *IL6* polymorphism at nucleotide −174, the CC genotype was significantly lower (3.3% vs 11.0%, respectively; OR = 0.36; 95% CI: 0.144–0.793) compared to HC group and the G allele was overrepresented in the LTBI group. *IL-6* levels did not differ significantly between the genotypes of the *IL-6* − 174G/C SNP in the HCs and patients.

### Multilocus genotype association with the risk of LTBI and development of TB

We also assessed whether genotype combinations (in *IFN-γ*, *TNF-α*, *IL-10*, and *IL-2*) for the mutations in these cytokines contribute to an increased risk of TB or LTBI. There were 15 possible combinations. There was a clear association between *IFN-γ* −874A and latent TB, especially in the combinations AAGT (OR = 1.70; 95% CI: 1.079–2.665), AGAG (OR = 9.25; 95% CI: 5.300–15.932), and AGGG (OR = 1.98; 95% CI: 1.125–3.505), whereas *IL-10* − 1082 was associated with an increased risk of PTB in the combination of AAAT (OR = 2.01; 95% CI: 1.252–3.219) and AGAG (OR = 1.41; 95% CI: 1.062–2.575; (Table 3).

### Dose effects of genes and gene–environment interactions

In addition to the multiplicative and additive models, we also analyzed the dose effects of the genotypes using the dominant and recessive models for each of the four genotypes and their interactions with BCG (Table 4). The effect of the *IFN-γ* +874 T allele was recessive for LTBI, whereas *IFN-γ* +874 T was dominant for PTB. The *TNF-α* and *IL-2* polymorphisms were not significant in these models (Table 4). BCG vaccination was found to compensate for the positive effect of the *IFN-γ* polymorphism and was associated with a reduced risk of LTBI in the additive model.

**Table 4:** **Dose effects of gene–gene and gene–environment interactions on susceptibility to LTBI and PTB**

**Cytokines**	**SNP position**	**Genotypes**	**LTBI.vs.HC**		**PTB.vs.LTBI**	
			**Gene**	Gene*BCG	**Gene**	Gene*BCG
IFNγ	(+874)T- > A	Additive	0.092	0.093	0.264	0.093
		Dominant	0.616	0.193	0.010*	0.117
		Recessive	0.003*	0.277	0.552	0.405
		Multiplicative	0.174	0.382	0.976	0.979
IL-10	(−1082)G- > A	Additive	0.015	0.909	0.540	0.466
		Dominant	0.485	0.837	0.208	0.507
		Recessive	0.880	0.775	0.098	0.709
		Multiplicative	0.423	0.749	0.134	0.411
TNFα	(−308)A- > G	Additive	0.845	0.722	0.945	0.873
		Dominant	0.995	1.000	0.995	0.999
		Recessive	0.072	0.326	0.144	0.700
		Multiplicative	1.000	0.999	0.995	0.994
IL2		Additive	0.815	0.909	0.437	0.485
		Dominant	0.485	0.837	0.845	0.613
		Recessive	0.881	0.775	0.253	0.223
		Multiplicative	0.423	0.749	0.577	0.397

Notes: HC, healthy controls; LTBI, subjects with latent tuberculosis infection; PTB, patients with pulmonary tuberculosis; INF-γ, interferon γ; IL-10, interleukin 10; TNF-α, tumor necrosis factor α; IL-2, interleukin 2.

*p < 0.05.

## Discussion

SNPs in several candidate genes have been linked to an increased risk of TB [12]. In this polymorphism association study, we investigated the significance of the relationship between several cytokine gene polymorphisms, their plasma products, and susceptibility to TB in a Chinese population.

The IFN-γ polymorphism is the best-studied polymorphism in terms of its association with TB disease sites and severity. However, previous reports have been contradictory insofar as the −874A allele was more common in patients with TB and the T allele was more common in the controls in Italian [13], South African [14], and Spanish populations [15], whereas in China, one study reported no association [16] while a later study of the same population showed an association between TB and the A allele [17]. Our results are consistent with the latter study. In the present report, we show that the A allele at *IFN-γ* −874A/T was significantly overrepresented in the populations of LTBI and PTB patients, with A allele frequencies 1.30-fold and 1.76 fold higher, respectively, than those in the HC. Significantly reduced *IFN-γ* production by stimulated PBMCs was observed in individuals with the A allele at *IFN-γ* −874. Similar genotype distributions and associations were observed for the t875 CA repeats (non-12/12) as at −874A/T in our study, and the non-12 CA repeats allele was absolutely linked to the −874 A allele in our population, as previously reported in a UK population. Because the −874 A allele corresponds to lower *IFN-γ* expression, the lower IFN-γ levels may impair the activation of macrophages, resulting in the development of TB. These observations indicate that the A allele at *IFN-γ −*874, or another nearby genetic variation linked to it, increases the host’s susceptibility to TB.

Because the modulation of the T-cell response by *IL-10* seems to influence the susceptibility of the host to TB infection [18], identifying the polymorphisms in the *IL-10* gene may be useful in predicting disease susceptibility. The frequency of the *IL-10* − 1082G allele in our population (4%) was similar to that in Korean (7.4%) [19] and Japanese (6.5%) populations [20], but was significantly different from that reported in Caucasians (48%) [21]. The present results also show that the genotype frequency of the *IL-10* − 1082A allele was significantly increased in TB patients compared with the LTBI subjects, especially when it was associated with the *IFN-γ* +874A allele (OR = 3.59; *p* = 0.045). This is consistent with the low levels of mycobacterial-stimulated *IFN-γ* and *IL-10* in PTB patients. In fact, there remains a discrepancy with the −1082 polymorphism in the *IL10* promoter, for which no association with TB was reported in Gambian [22] and Korean populations [23]. However, an association between the *IL-10* − 1082A allele and TB was observed in an Italian (Sicilian) [24] population, and GA heterozygosity was associated with PTB in Cambodia [25]. These observations may reflect ethnic-specific genetic variations or suggest that other more-distal promoter elements are involved.

A number of studies have investigated the association between the *TNF-*α 308 (rs1800629 G/A) polymorphism and susceptibility to TB in different populations. However, the association between TB with *TNF-α* differs with ethnicity, and many of these studies have produced inconsistent results [26]. In the present study, a significant negative association between TB and the *TNF-α* 308 GA genotype and a significant positive association with the GG genotype were found, and the G allele was carried by 90% of patients and by 85.8% of controls. The A allele is associated with high *TNF-α* production and the G allele is associated with low *TNF-α* production. The high frequency of the genotype associated with increased protein production may be attributable to selection during the course of evolution, increasing the frequency of the most advantageous genotype. Our data indicate that *TNF-α* production was reduced in our PTB patients. Although the study by Scola et al. demonstrated a reduction in the low-producing −308 G/G *TNF-α* homozygous individuals in the affected group [24], and the study of Bikmaeva et al. revealed an association between the high-producing A allele and a higher risk of PTB [27], although several studies have failed to confirm the association between *TNF-α* and TB [28].

In the present study, we also analyzed several other candidate gene polymorphisms and their association with the susceptibility to PTB or LTBI. We found a significant negative association between the −330 TT genotype of *IL-2* and PTB; the T allele was significantly overrepresented in the LTBI group and the HC relative to the PTB group. This suggests that the GG haplotype is associated with a susceptibility to PTB. Although translation studies have demonstrated the increased activity of the G allele, allele expression studies have shown higher expression of *IL-2* in the PBMCs of individuals with the *IL-2* − 330 TT genotype than in those of individuals with the GG genotypes, suggesting that the *IL-2* promoter activity is also influenced by other polymorphisms, probably +160 T/G [29]. No significant association between TB and IL-4 levels was observed for the VNTR polymorphism. The results of the present study do not preclude the possible influence of the VNTR polymorphism on the *IL-4* response because *IL-4* was undetectable in most of the subjects tested, which could be attributable to the downregulation of the Th2 responses by the *IFN-γ* secreted in response to the antigens used. In our study, the *IL-6* − 174 CC genotype was significantly negatively associated with the LTBI group and with the reduced production of *IL-6*. In previous studies, significant levels of *IL-6* were shown to be produced in response to Mtb infection. Human and murine macrophages secrete *IL-6* in response to Mtb *in vitro* [30], and elevated concentrations of *IL-6* are present in the plasma of patients with TB [31]. It has also been reported that the induction of *IL-6* activity by Mtb inhibits *IFN-γ* signaling, so the bacterium evades eradication by the cellular immune response [32]. We have reported that the *IL-12* AA genotype is associated with LTBI susceptibility. This is consistent with reports from Japan and France, where polymorphisms in the *IL-12* receptor β1 gene (*IL-12RB1*) are known to influence the susceptibility to and severity of TB [33].

A consensus seems to be emerging that the combined effects of several cytokine SNPs may play a crucial role in susceptibility to LTBI and TB diseases [29]. The combination of the *IFN-γ* polymorphism with other cytokine polymorphisms was most effective in predicting LTBI. This observation is consistent with the recently described functions of *IFN-γ* in a mouse model, in which mice with a disrupted *IFN-γ* gene were unable to control Mtb infection [34]. The combination of *IL-10* polymorphisms with other cytokine polymorphisms may predict a susceptibility to TB. These results also substantiate an earlier report [35] that implicated *IL-10* in the reactivation of TB in humans and in TB disease in a mouse model. A recently published meta-analyses reinforced the critical importance of *IFN-γ* +874 T/A as a genetic marker of TB resistance [8], and stressed that although *IL-10* has a specific effect on the form and severity TB, it does not affect susceptibility *per se*. Our results are consistent with these findings. BCG vaccination cannot be ignored [36]. However, we found no evidence that BCG vaccination interacts with gene polymorphisms in affecting susceptibility to TB.

In this study, all cases of TB disease were included, but the majority of patients had PTB. Therefore, it was not possible to demonstrate an association between the system involved and cytokine polymorphisms. A larger series is required to test the possible associations between cytokine gene polymorphisms and organ preference in TB, and the clinical severity of the disease.

## Conclusions

Single-nucleotide functional polymorphisms in cytokine genes display variable associations with LTBI and active TB disease. Combinations of cytokine SNPs with the *IFN-γ* +874 T/A SNP markedly influence the severity and outcome of TB. Our results may reflect the polygenic aspects of a predisposition to severe and active TB.

## Methods

### Study subjects

Two case–control studies consisted of 120 patients diagnosed with PTB (patient group), 240 with LTBI (the control group for the patient group, and the case group for the HC group), and 480 HC individuals (control group). The subjects were recruited during the routine investigation of individuals in contact with TB in seven districts of Shanghai between 2009 and 2010. The subjects in the patient group were bacteriologically confirmed as TB patients infected with Mtb. None of the patients reported infection with human immunodeficiency virus. LTBI was identified in the contacts of TB patients, and had no TB-related symptoms but a positive T-SPOT.*TB* test. The subjects in the HC group were also recruited from the contacts of TB patients, with no TB-related symptoms or previous history of TB, and a negative T-SPOT.*TB* test*.*

### *T-SPOT.*TB *assays*

T-SPOT.*TB* assays were performed according to the manufacturer’s instructions (Oxford Immunotec, Oxford, UK). Briefly, PBMCs were isolated from whole blood by centrifugation with Lymphocyte Separation Medium (Ficoll 400), washed twice, and incubated with ESAT-6 and CFP-10 antigens for 16–20 h (37°C, CO_2_) in an enzyme-linked immunosorbent spot assay. The spots were counted. The T-SPOT.*TB* result was positive when the number of spots in the test well (against at least one of the two tested antigens) was at least six, if the negative control had fewer than five spots, or twice the number of spots in the negative control wells, if the negative control had more than six spots. This cutoff was predefined in the manufacturer’s instructions.

### Whole blood stimulation assays

The stimulated whole blood (WB) culture assay used to assess the cytokine profiles has been described in detail previously [37]. Briefly, heparinized blood was diluted 1:11 with sterile RPMI 1640 tissue culture medium containing 100 units/ml penicillin/100 mg/ml streptomycin and 2 mM L-glutamine (Sigma Chemical Co., St. Louis, MO, USA). Within 2 h of collection, diluted WB (900 ml/well) was stimulated with Mtb culture filtrate proteins (5 mg/ml) in a 24-well tissue culture plate. The supernatants were collected from the wells at varying intervals.

### Cytokine assessment

The cytokines in the supernatants were assessed using pairs of monoclonal antibodies, as described in detail previously [38]. Dose–response curves were constructed for each individual plate. The supernatants were serially diluted and the optical density (OD) readings in the linear ranges of the dose–response curves were used to calculate the concentrations. The final concentrations (pg/ml) were obtained by multiplying the values by the dilutions at which the ODs were read. The sensitivity range of cytokine detection was 7.5–1000 pg/ml and was similar to that reported by the manufacturer.

### Molecular methods

Genomic DNA was extracted from blood samples with the Puregene Genomic DNA Isolation Kit (Gentra Systems, Minneapolis, MN, USA), according to the manufacturer’s instructions. Primers were purchased from MWG-Biotech AG (Ebersberg, Germany). *IFN-γ* (rs2430561), *IL-10* (rs1800896), and *TNF* (rs1800629) were genotyped with amplification-refractory mutation system–polymerase chain reaction (ARMS-PCR), with human growth hormone or bactin primers as the internal controls to check the accuracy of the PCR reactions. *IL-6* (rs1800795) was genotyped with tetra-ARMS-PCR. The amplified products (5 ul, undiluted) were monitored with electrophoresis on agarose gel prepared in Tris–acetate ethylene-diaminetetraacetic acid (TAE) buffer containing 10 mg/ml ethidium bromide. The product bands were visualized on a UV transilluminator and photographs were taken for genotype interpretation.

### Confirmatory sequencing methodology

DNA sequencing was applied to a subset of samples (10%–15% for each SNP) to confirm the genotypes identified with ARMS and tetra-ARMS-PCR. The primers used for the sequencing reactions for *IFN-γ* and *IL-10* were designed using the Lasergene version 7.0 software (DNASTAR, Madison, WI, USA), and the primers for *TNF* and *IL-6* were designed using the web-based BatchPrimer3 software. Sequence data produced with the ABI 3130xl Genetic Analyzer were reviewed for confidence levels with the ABI Sequence Scanner software, and the sequencing results were analyzed with pairwise alignments of the sequences with ClustalW version 1.83. The allelic specificities of the cytokine gene SNPs were determined by comparison of the PCR results with the nucleotide sequencing results for the alleles. Concordance of > 95% was observed in all cases.

### Statistical analysis

Data were analyzed with SPSS for Windows version 16 (SPSS Inc., Chicago, IL, USA). The cytokine levels were expressed as means ± standard error. Hardy–Weinberg proportions were determined by applying the equation (p^2^ + 2pq + q^2^). All polymorphisms were tested for Hardy–Weinberg equilibrium separately in the patient and control groups by comparing observed and expected numbers using the χ^2^ test. The distributions of the cytokine gene polymorphisms were compared between the different study groups with the χ^2^ test or Fisher’s exact test if a cell was below 5. Values of *p* were smaller than 0.05 were considered significant. Odds ratios (OR) and 95% confidence intervals (CI) were also calculated. A logistic regression analysis was used to determine the effects of age and sex on the genotypes. Combinations of the *IFN-γ* +874 TA genotype with the *IL10* 21082 AG, *TNF* −308 GA, or *IL-6* 2174 GC genotype were used to determine the two-gene combination effects. Nine possible genotypes were compared between the controls and PTB patients using Pearson’s χ^2^ or Fisher’s exact test.

### Ethics committee approvals

The study protocol was approved by the Ethics Committee of the School of Public Health, Fudan University, and written informed consent was obtained from all study participants.

## Abbreviations

TB: Tuberculosis
Mtb: Mycobacterium tuberculosis
IFN-γ: Interferon-gamma
IL: Interleukin
LTBI: Latent tuberculosis infection patients
PTB: Pulmonary tuberculosis
HC: Healthy control
BCG: Bacille Calmette– Guérin
